# Reconstruction and Validation of RefRec: A Global Model for the Yeast Molecular Interaction Network

**DOI:** 10.1371/journal.pone.0010662

**Published:** 2010-05-14

**Authors:** Tommi Aho, Henrikki Almusa, Jukka Matilainen, Antti Larjo, Pekka Ruusuvuori, Kaisa-Leena Aho, Thomas Wilhelm, Harri Lähdesmäki, Andreas Beyer, Manu Harju, Sharif Chowdhury, Kalle Leinonen, Christophe Roos, Olli Yli-Harja

**Affiliations:** 1 Department of Signal Processing, Tampere University of Technology, Tampere, Finland; 2 Medicel Ltd., Espoo, Finland; 3 Institute of Food Research, Norwich, United Kingdom; 4 Department of Information and Computer Science, Helsinki University of Technology, Espoo, Finland; 5 Biotechnology Center, Technische Universität Dresden, Dresden, Germany; Universidade de Sao Paulo, Brazil

## Abstract

Molecular interaction networks establish all cell biological processes. The networks are under intensive research that is facilitated by new high-throughput measurement techniques for the detection, quantification, and characterization of molecules and their physical interactions. For the common model organism yeast *Saccharomyces cerevisiae*, public databases store a significant part of the accumulated information and, on the way to better understanding of the cellular processes, there is a need to integrate this information into a consistent reconstruction of the molecular interaction network. This work presents and validates RefRec, the most comprehensive molecular interaction network reconstruction currently available for yeast. The reconstruction integrates protein synthesis pathways, a metabolic network, and a protein-protein interaction network from major biological databases. The core of the reconstruction is based on a reference object approach in which genes, transcripts, and proteins are identified using their primary sequences. This enables their unambiguous identification and non-redundant integration. The obtained total number of different molecular species and their connecting interactions is ∼67,000. In order to demonstrate the capacity of RefRec for functional predictions, it was used for simulating the gene knockout damage propagation in the molecular interaction network in ∼590,000 experimentally validated mutant strains. Based on the simulation results, a statistical classifier was subsequently able to correctly predict the viability of most of the strains. The results also showed that the usage of different types of molecular species in the reconstruction is important for accurate phenotype prediction. In general, the findings demonstrate the benefits of global reconstructions of molecular interaction networks. With all the molecular species and their physical interactions explicitly modeled, our reconstruction is able to serve as a valuable resource in additional analyses involving objects from multiple molecular -omes. For that purpose, RefRec is freely available in the Systems Biology Markup Language format.

## Introduction

Systems biology aims at producing information about system level phenomena in cells. Therefore, knowledge about cellular components, their interactions, and their state in different conditions needs to be collected and integrated. Given the large amount of information produced by the advanced measurement technologies in life sciences, it is noteworthy that no network reconstruction integrates the information globally and consistently, even not for the common model organism yeast (*Saccharomyces cerevisiae*).

Exploring data from any single molecular -ome alone (such as genome or proteome) only enables restricted systems understanding. A more holistic view can be obtained through extensive integration of molecular species and interactions related to multiple -omes. These kinds of integrative reconstructions of global cellular networks find many applications, for example, in the building of predictive models of cellular phenomena and in the description of measurement data in the context of a system. Explicit representation of all molecular species and interactions enables continuous integration of more data originating from heterogeneous sources. For example, data from large genomic variation studies of single nucleotide polymorphisms and copy number variations can be associated with data obtained by transcriptome quantization or protein profiling. Thus, network reconstructions which are general enough and not tailored for any specific purpose, can be re-used in various studies.

Traditionally, there has been no unifying information model conceptualizing and organizing the diverse information in the field of molecular biology [1],[2]. Databases, typically storing focused information about a single molecular -ome, use a rather narrow information model to represent the objects and their properties. The variability of information models originates from the diverse needs of the respective research and from the difficulty to coordinate the development of a comprehensive information model in a rapidly changing experimental field. Such a lack of a more general information model hampers the integration across research domains. Integration of data from different databases introduces problems like differences in object definitions, names, and data versions. The larger the source databases are and the more complex information they contain, the more difficult the integration becomes. Therefore, there is a need for automated network reconstruction tools which are capable of integrating information from multiple databases covering multiple molecular -omes.

Metabolic network reconstructions are obtained by automated methods which work mainly by using genomic sequence information [3]–[8], but also by taking into account additional data on e.g. mRNA co-expression [9], phylogenetic profiles [10], gene clustering on chromosomes, protein fusion [11], and knowledge about other thoroughly curated metabolic networks [12],[13]. There are also promising new methods as mass spectrometric metabolome mapping, metabolite correlations, genetical genomics of metabolism, and flux measurements [14]. One of the largest reconstruction collections established using the automated reconstruction methods is BioCyc [13] that currently covers more than 370 organism-specific databases. Interestingly, the BioCyC collection does not contain a reconstruction for *S. cerevisiae*. Instead, the first genome-scale draft reconstruction on yeast metabolic network [15] has been iteratively refined with the help of literature information, wet-lab experiments, and computational modeling [16]–[19]. The genome-scale metabolic reconstructions describe a fundamental but still a limited part of the entire molecular interaction network of yeast. For example, the most recent genome-scale metabolic reconstruction [19] contains 12.5% of all yeast genes documented in the Ensembl database [20] and 10.3% of all the essential genes [21],[22]. Thus, comprehensive systems understanding of the yeast cell can be promoted by integration of additional data.

One of the main properties of a comprehensive information model is the use of so called reference objects which can be identified unambiguously. For example, common or systematic names of genes may change or become obsolete over the years and therefore they do not serve for this purpose. In contrast, the primary sequence is the most fundamental property of genes, transcripts, and proteins, and thus their equivalency can be exploited for the identification of the reference objects within genomic, transcriptomic, and proteomic databases (see, e.g., [23]–[25]). We used this reference object approach by integrating molecular species and interactions from different source databases into a single network reconstruction called RefRec, thereby introducing a reference object based reconstruction for the yeast molecular interaction network. The RefRec reconstruction is available in the BioModels database [26] with the accession number MODEL3883569319. Metabolites in the reconstruction were obtained from a single source database (Kyoto Encyclopedia of Genes and Genomes, KEGG) [27]–[29] in order to avoid namespace-related issues. Interestingly, an approach complementary to ours has recently been presented [19]. Reference objects are there introduced for metabolites – by identifying them using Simplified Molecular Input Line Entry System (SMILES) [30] and International Chemical Identifiers (InChI) [31],[32] – while genes and proteins are imported from single source databases.

**Figure 1:**
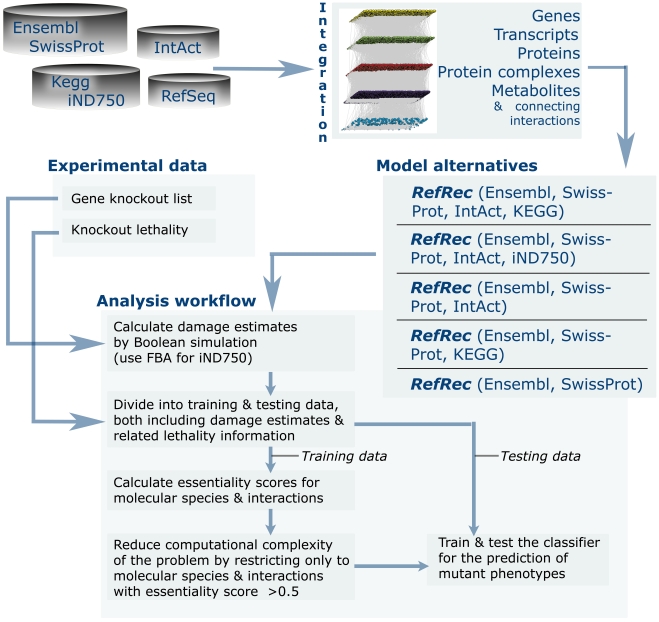
Overview to the presented analysis. The RefRec reconstruction was integrated from selected databases. The reconstruction was converted to a set of model alternatives which were used to assess the importance of different molecular –omes for accurate phenotype prediction (from top to down: RefRec; RefRec with KEGG replaced by iND750; RefRec without a metabolic network; RefRec without a protein-protein interaction network; RefRec without a metabolic network and a protein-protein interaction network). All the model alternatives were analyzed using a single analysis workflow that first estimates gene knockout damages in mutant strains and then trains a computational classifier to predict the mutant viability.

In this work, we aimed to build and validate a global reconstruction for the yeast molecular interaction network. The RefRec reconstruction was obtained by integration of major biological databases with the help of unique reference objects. The reconstruction explicitly models the structure of protein synthesis pathways, a metabolic network, and a protein-protein interaction network. The provided database cross-references make it possible to further extent the reconstruction with e.g. the knowledge of gene regulation and signal transduction networks. In order to validate the reconstruction and to demonstrate its biological relevance for various applications, we exploited experimental growth phenotype information for hundreds of thousands yeast strains carrying genetic mutations. We showed that the phenotype can be best predicted while taking all types of molecular species and interactions into the consideration, and that the RefRec reconstruction is capable for predicting the lethality of gene knockouts affecting a substantial variety of biological processes. Figure 1 outlines the analysis approaches applied in this research.

## Results

### Characteristics of the reconstruction

The global molecular interaction network for yeast was reconstructed by integrating information from seven large molecular databases. All the yeast related molecular species (genes, transcripts, proteins, protein complexes, and metabolites) and interactions were obtained from the databases and integrated into a consistent and non-overlapping network reconstruction. The total number of objects in RefRec is 67,228 which are divided up according to their types in Table 1. The merge of proteins originating from three databases (Ensembl, RefSeq, and UniProt) resulted in a greater number of unique proteins than specified in any single protein database. Metabolites and protein complex assembly interactions were imported from single source databases (KEGG and IntAct, respectively) which prevented the contradictions in naming conventions. The reconstruction was supplemented by such molecular species and interactions which were not described in the used databases but whose existence is evident in a complete physical interaction network. For example, transcription processes are not explicitly represented in any database, but in a physical interaction network they have their specific role. Therefore, some objects documented in databases (i.e., genes, transcripts, and protein-protein interactions) were used as source information to instantiate the non-documented objects (i.e., transcriptions, translations, and protein complexes).

**Table 1:** Origin and number of the molecular species and interactions in the reconstruction.

Database	Gene	Transcription	Transcript	Translation	Protein	Protein complex assembly interaction	Protein complex	Metabolic reaction	Metabolite
**Information for the reconstruction structure**									
Ensembl [20]	6,648 (o, r)		6,647 (o, r)		6,617 (o, r)				
RefSeq [23]					5,819 (o, r)				
UniProt [48]					6,126 (o, r)				
KEGG COMPOUND [27]									812 (o)
KEGG GLYCAN [28]									55 (o)
KEGG REACTION [29]								2,304 (o)	822 (x)
IntAct [49]					5,161 (x)	15,366 (o)			
Instantiated objects		6,648		6,647			15,366		
**Cross-references to additional databases**									
PubChem [50]									799
ChEBI [51]									591
**Unique totals**	6,648	6,648	6,647	6,647	6,780	15,366	15,366	2,304	822
**Unique molecular species**									36,263
**Unique interactions**									30,965
**Unique objects**									67,228

RefRec represents physical interactions between the molecular species and explicitly reconstructs all the involved objects. Objects originating from the databases (o), reference objects obtained by sequence comparisons (r), and objects represented in databases by name-based cross-references to other databases (x).

**Figure 2:**
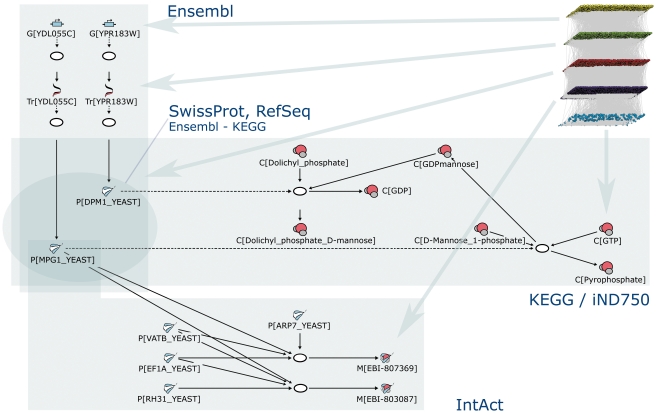
Structural details of RefRec. The entire reconstruction is visualized in top right where different types of molecular species are grouped to layers (from top to down: genome, transcriptome, proteome, protein complexes, and metabolome) and interactions are depicted by the connecting edges. Structural details are shown from each of the layers as follows: Ovals represent interactions and the other nodes represent molecular species of different types. The Ensembl database provides source information for protein synthesis pathways. Two genes (labeled as G[•]) are transcribed to transcripts (Tr[•]) and further translated to proteins (P[•]). The KEGG database provides knowledge for the metabolic network including metabolites / compounds (C[•]) and metabolic reactions. The enzymatic activity of proteins is described by the Swiss-Prot ENZYME database that is used to connect the proteins to the metabolic reactions. The protein-protein interaction network including the protein complex assembly interactions and the protein complexes / macromolecular complexes (M[•]) is based on the IntAct database. The IntAct database does not provide information about enzymatic activity for protein complexes, and therefore protein complexes do not catalyze any interaction in the reconstruction. A dashed arrow presents the control of an interaction by a molecular species. A solid arrow presents material flow.

The reconstruction represents the molecular species and interactions at protein synthesis pathways (from genes to proteins), a metabolic network, and a protein-protein interaction network. Figure 2 illustrates the structure of RefRec. In the protein synthesis pathways, all the genes control their associated transcription processes producing transcripts. Further, transcripts control their translation processes producing the proteins. There are two genes (gene loci *YEL076C-A* and *YLR464W*) whose transcripts have identical nucleic acid coding sequences, and were therefore considered as a single transcript. All transcripts have a known protein product but, on the other hand, 163 proteins appear without a transcript (these proteins are documented in RefSeq and/or UniProt database but not in Ensembl that provides information about the protein synthesis pathways). Proteins have a central role in the network as they catalyze metabolic reactions (650 proteins) and they are used as substrates in protein complex assembly interactions (5,161 proteins). While the median number of interactions per protein is four in our global reconstruction, the most highly connected proteins (HSP72, HSP75, EF1A, and UBC7) take part in more than 500 interactions. The number of proteins per protein complex assembly interaction gives a median of two and a maximum of 99 (the subunits of the 26S proteasome and the proteasome interacting proteins). Especially highly connected biomolecules can be also found in metabolism. The most connected metabolite, water, acts in 527 metabolic reactions as a substrate and in 239 reactions as a product. Common energy carriers, such as adenosine triphosphate (ATP) and nicotinamide adenine dinucleotide (NADH), are among the other highly connected metabolites. In median, metabolites participate in four metabolic reactions as a substrate or a product.

### Gene knockout damages

Based on our established global molecular interaction network reconstruction we estimated damages caused by gene knockouts. Typically, a gene knockout results in the blockage of the corresponding transcription process, which results in the blockage of the transcript, and so forth. Depending on the network structure, the blockage may propagate further to metabolism and protein-protein interactions. Taken together, the set of the blocked interactions and molecular species describes the damage caused by the gene knockout to the molecular interaction network.

We estimated the network damage using two alternative approaches. First, the whole RefRec reconstruction was translated to a Boolean model, and the damage propagation of a knockout was simulated using a Boolean simulation algorithm (see Materials and Methods for details). Second, the metabolic part of RefRec was replaced by an alternative metabolic reconstruction iND750 [16]. The replacement included metabolic reactions, metabolites, and simplified direct regulation of metabolic reactions by the genes. Again, the damage in the non-metabolic part of the reconstruction was estimated using the Boolean method but a flux balance based method [33] was used to estimate the damage within the metabolic iND750 reconstruction. The damages estimated based on the iND750 reconstruction are called unconditional because we assumed aerobic cultivation conditions and a complex medium. The same knockouts assuming any reduced medium would at least contain the same unconditional damage, in addition to a possible conditional damage.

**Figure 3:**
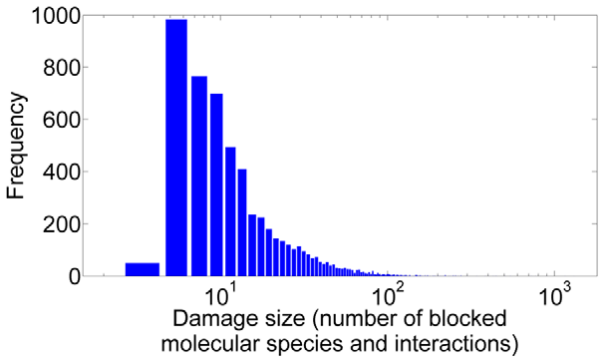
Size distribution of the damage estimates for single gene knockouts. The damages are estimated by Boolean analysis of the RefRec reconstruction.

As depicted in Table 2, the damages for a genome-wide set of single gene knockouts [22] were estimated to propagate to all the five molecular -omes but with varying probability. For example, a gene knockout always resulted in the blockage of the corresponding transcription, transcript, and translation. However, proteins were not damaged in all the cases because a few proteins have alternative routes of production. Protein complex assembly interactions were typically affected, the average number of blocked assembly interactions being around ten per knockout but with large variation. Metabolic reactions and metabolites had a substantially lower probability to be affected by a gene knockout because the number of metabolic enzymes is considerably smaller than the number of proteins involved in the protein-protein interaction network. The total number of blocked molecular species and interactions within the reconstruction varied between 4 and 1797 (cf. Figure 3). The damages propagated more frequently to the KEGG reconstruction than to the iND750 reconstruction. However, the analysis of the iND750 reconstruction resulted in larger damage estimates than the analysis of the KEGG reconstruction.

**Table 2:** Statistics about damage estimates for single gene knockouts.

Object type	Analysis method	Damage probability	Mean number of blocked objects in a damage (+SD)
Gene	B	1	1 (0)
Transcription	B	1	1 (0)
Transcript	B	1	1 (0)
Translation	B	1	1 (0)
Protein	B	0.99	1 (0)
Protein complex assembly interaction	B	0.81	10.6 (26.7)
Protein complex	B	0.81	10.6 (26.7)
Metabolic reaction (KEGG)	B	0.1	4 (5.1)
Metabolite (KEGG)	B	0.02	3 (2.8)
Metabolic reaction (iND750)	FBA	0.05	12.2 (25.1)
Metabolite (iND750)	FBA	0.04	10.8 (20.1)

Damages estimated using Boolean simulation (B) or flux balance analysis (FBA).

### Unconditionally essential molecular species and interactions

**Figure 4:**
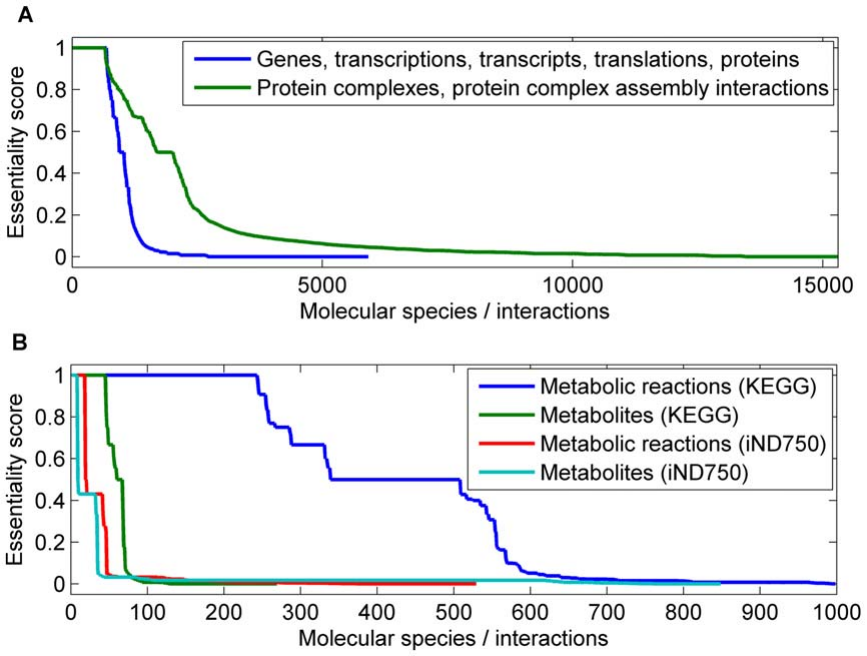
Essentiality scores for molecular species and interactions. The essentiality score indicates the relative frequency of the experimentally observed inviable phenotype under the cases where the examined molecular species or interaction was blocked. The objects are sorted in the descending order of the essentiality score. In panel A, the graphs indicated in the legend are merged because of negligible differences between them. In panel B, 1,005 metabolic KEGG reactions having the score value zero are not shown.

All types of molecular species and interactions include objects that can be considered unconditionally essential for viability. This was shown with the help of an essentiality score that we calculated for each of the molecular species and interactions (commonly called as ‘objects’). The score was defined as the fraction of experimentally validated lethal knockouts blocking a particular object within the set of all experimental knockouts that block this object. A possible bias may be introduced to the score because experimental knockouts are not evenly nor comprehensively distributed to the network but they concentrate on specific parts of it. For example, if only a single knockout indicating essentiality for an object is available, essentiality will be inferred. But as more knockouts are added, the chances of inferring essentiality will drop, simply because some of the new experiments might not infer essentiality. Thus, the fewer knockouts block an object, the more biased its score may be. In the score calculation we used experimentally obtained viability information and our computational damage estimates of ∼590,000 mutant yeast strains carrying single, double, or triple gene mutations (see the description of the experimental data in Materials and Methods and Table 3). As illustrated in Figure 4, all types of molecular species and interactions include objects whose blockage is related to the inviable phenotype either invariably (i.e. the objects have essentiality score one and they are now called unconditionally essential) or on some occasions (essentiality score greater than zero but less than one).

**Table 3:** Experimental yeast mutant data used in the analysis.

Data source	Number of deleted or mutated genes	Viable mutant strains	Inviable mutant strains	Unique totals for data sources
[22]	1	4,670	1,100	5,770
SGD	2	7,063	8,996	15,080
[36]	2	578,663	2,323	573,605
[36]	3	178	12	190
**Unique totals for phenotypes**		580,188	10,108	

Viable mutant strains consist of normal and slow growing mutant strains. The unique totals are shown as the information in the data sources partly overlaps.

**Figure 5:**
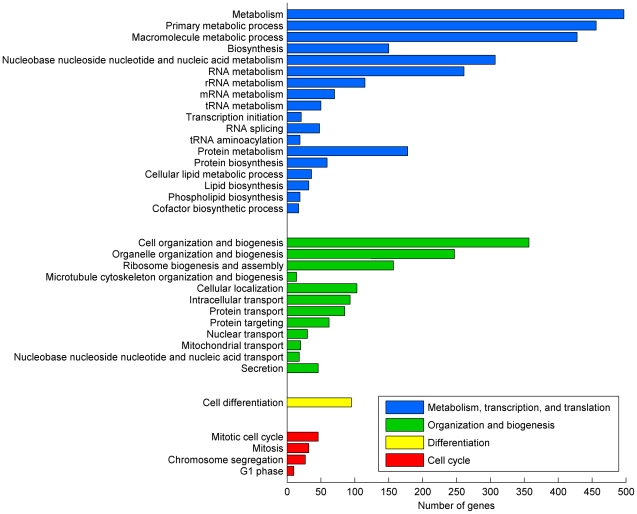
Gene Ontology enrichment analysis for the unconditionally essential genes. The figure presents a representative partial list of the significantly enriched GO Biological Process categories associated with the unconditionally essential genes. Note the wide scope of the term metabolism in Gene Ontology where metabolic processes are associated with all types of molecular species.

The essentiality score detects 689 unconditionally essential genes. For these genes, the used experimental dataset gives no evidence about cultivation conditions or parallel additional gene mutations that would rescue the cell from the lethal effects of their knockouts (96% of these unconditionally essential genes have also been documented within the set of 1,100 essential genes in a single gene knockout study [22]). In order to outline the most important processes for the cellular viability, we mapped the unconditionally essential genes to the biological processes described in Gene Ontology (GO, accessed June 14^th^ 2007) [34]. Table S1 lists the unconditionally essential genes together with the biological processes that were significantly enriched (according to the two-sided Fisher's exact probability test with P<0.05; all yeast genes as the reference set) and contained at least ten unconditionally essential genes. Figure 5 presents a representative partial list of the significantly enriched processes. Most of the enriched processes can be categorized under four general themes also shown in Figure 5. The enriched processes represent central cellular phenomena and they involve all types of molecular species and interactions.

The 658 unconditionally essential protein complexes included well known necessary protein complexes, such as RNase MRP that cleaves the yeast pre-rRNA, and Tim23 involved in protein translocation. In addition, hundreds of protein pairs mainly detected by the yeast two-hybrid technique were found unconditionally essential. The synthetic lethal gene pairs identified e.g. in [35],[36] manifest themselves as the production blockage of these bimolecular protein complexes.

In the KEGG metabolic reconstruction, the number of unconditionally essential metabolic reactions and metabolites was 243 and 45, respectively. These objects are involved in necessary metabolic processes, such as energy metabolism (e.g. glucose-6-phosphate isomerase), riboflavin metabolism (e.g. FMN and riboflavin), and fatty acid biosynthesis (e.g. acetoacetyl ACP). In contrast, the metabolic reconstruction iND750 contained 18 unconditionally essential reactions and 8 unconditionally essential metabolites. Also they are mainly related to vital processes like energy metabolism (e.g. citrate synthase), biosynthesis of amino acids, purines, and pyrimidines (e.g. dihydrofolate reductase), and transport of metabolites (e.g. glutamine transport). The majority (10) of the unconditionally essential reactions identified using iND750 were also identified using KEGG while the unconditionally essential metabolites identified using the two reconstructions did not overlap. The differences in the results between KEGG and iND750 are discussed in Discussion.

### Prediction of growth phenotypes

In addition to the explorative analysis of the unconditionally essential molecular species and interactions, we used RefRec to predict the growth phenotypes for the ∼590,000 single, double, and triple gene knockout yeast mutants. We trained a support vector machine classifier to produce knockout lethality predictions based on the damage estimates of the knockouts. As described in Materials and Methods, the essentiality score was calculated for a training data set and used to reduce the complexity of the problem prior to the classifier training. In each of the following analyses, the classifier was trained and tested ten times with randomly chosen samples in order to implement the randomized hold-out validation. The prediction performance was evaluated using the true positive rate (defined as the fraction of inviable phenotypes correctly predicted) and the false positive rate (the fraction of viable phenotypes incorrectly predicted as inviable).

**Figure 6:**
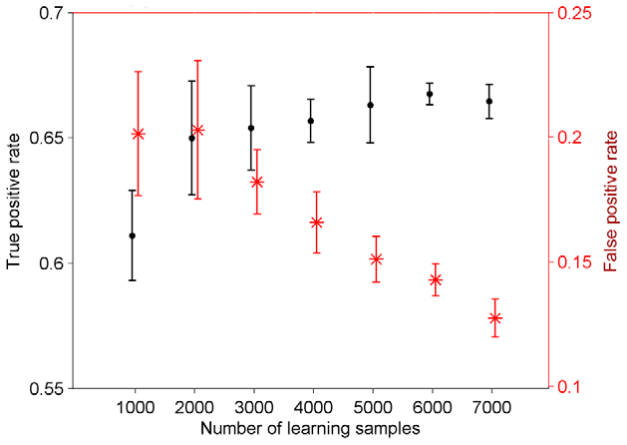
Prediction performance of the inviable phenotype affected by the number of training samples. In each case, 1/6 of the training samples are inviable. The average in the repeated analysis is represented using the central mark, and the whiskers represent the standard deviation excluding outliers.

**Figure 7:**
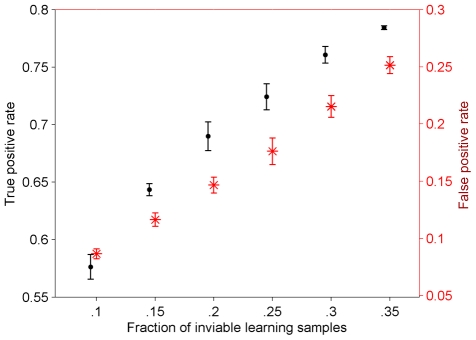
Prediction performance of the inviable phenotype affected by the fraction of inviable training samples. The total number of training samples is fixed to 7,000, and the test varies the number of inviable training samples within them. The average in the repeated analysis is represented using the central mark, and the whiskers represent the standard deviation excluding outliers.

As depicted in Figures 6 and 7, the sensitivity of detecting the inviable phenotype and the prediction confidence were affected by the total number of training samples, and especially the fraction of inviable phenotypes in them. Depending on these two parameters, the classification was able to predict correctly up to 79% of the inviable phenotypes while the false positive rate varied between 8% and 25%.

**Figure 8:**
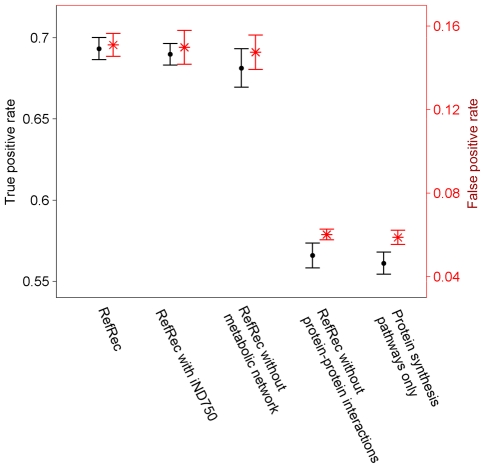
Prediction performance of the inviable phenotype affected by the available molecular -omes. RefRec with iND750 refers to the modified RefRec where the metabolic damage is estimated using the iND750 reconstruction instead of the KEGG reconstruction. The average in the repeated analysis is represented using the central mark, and the whiskers represent the standard deviation excluding outliers.

We further examined the significance of different types of molecular species and interactions for the successful lethality prediction. In this case, the number of learning samples was set to 7,000, the fraction of inviable learning samples was set to 0.2, and different types of molecular species and interactions were excluded from RefRec in turn prior to the classification. As depicted in Figure 8, the change of the metabolic reconstruction from KEGG to iND750 had practically no effect to the prediction performance. A more notable difference took place when the classifier was trained without damage estimates from any metabolic reconstruction. The damage estimates related to the protein-protein interaction network had the greatest effect to the true positive rate. The lowest fraction of correctly predicted inviable phenotypes was obtained when the damage estimates of both the protein-protein interaction network and the metabolic network were not available for the classifier training, i.e. the training was based only on damage estimates of protein synthesis pathways.

**Figure 9:**
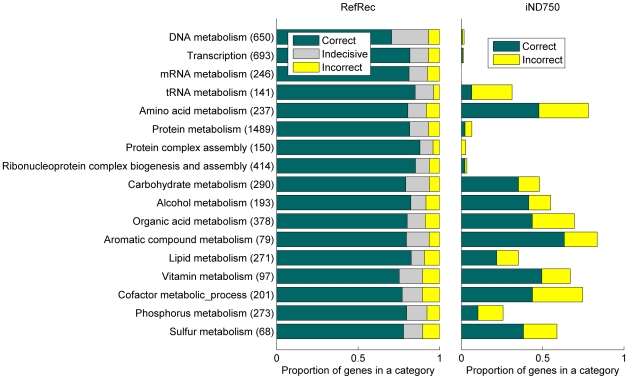
Growth phenotype prediction performance in relation to damage involvement in various biological processes. The selected Gene Ontology categories describe biological processes working with multiple molecular -omes and covering a large number of genes (given in parentheses). For the both reconstructions, bar lengths represent the number of single gene knockout predictions in proportion to the total number of genes in a category. The predictions with RefRec are produced using the unconditional damage estimates (this study) while the predictions with iND750 are produced using cellular growth estimates under seven cultivation conditions [16]. Note the more restricted scope of the term metabolism in RefRec and iND750 in comparison to Gene Ontology that associates metabolic processes with all types of molecular species.

The coverage and phenotype prediction performance of RefRec is illustrated through Gene Ontology that provides a holistic view to all the biological processes, molecular functions and cellular locations. In gene knockout phenotype prediction, a comprehensive reconstruction should in particular involve the biological processes essential for the cell. For example, the representation of the pentose-phosphate shunt in iND750 provides means to correctly predict the growth phenotype for the knockout of *YPR074C* (transketolase 1) under several cultivation conditions ([16], Supplementary material). In contrast, biological processes imperfectly represented in a reconstruction may result in prediction failures. In the case of iND750, this is reflected by the fact that the involvement of a gene in a non-metabolic process has been reported as the major reason for false predictions [16]. Figure 9 summarizes the coverage of selected GO categories by the genes represented in RefRec and iND750. While RefRec covers all the genes in all the categories, iND750 involves them only partially as it represents 11% of the yeast genes. In addition, Figure 9 shows the success of phenotype predictions for mutants carrying a knockout gene involved in a given category. A prediction based on RefRec was considered correct if most the repeated classification analyses (using the same training samples as in Figure 8) suggested the correct phenotype, and incorrect if most them suggested the incorrect phenotype. The success of predictions was relatively independent on the examined process, and on average 81% of the mutant phenotypes were correctly predicted per category. The predictions were best for the genes related to tRNA metabolism and protein complex assembly and worst for the genes related to vitamin, cofactor, and sulfur metabolism. In contrast, the prediction success depends significantly on the damaged process in the case of iND750 that was analyzed in an earlier study under seven cultivation conditions with the cell biomass production as the phenotype estimate [16]. There, the phenotype was best predicted for the genes involved in aromatic compound metabolism and vitamin metabolism. On the other hand, most of the predictions related to tRNA metabolism were incorrect, and in the case of six categories the phenotype could be predicted only for a small proportion of knockouts because the needed parts of the molecular interaction network were not represented by the reconstruction.

## Discussion

The presented global reconstruction RefRec summarizes most of the current knowledge of the yeast molecular interaction network. The network explicitly represents all the known genes, transcripts, proteins, metabolites, and protein complexes together with transcriptions, translations, metabolic reactions, and protein-protein interactions. The information about the genome, transcriptome, proteome, and metabolome may be considered relatively comprehensive because the complete yeast genome has been available since 1996 [37] and the yeast metabolism has been intensively studied (see, e.g. [38]). The information about the protein-protein interaction network remains still incomplete, as it is estimated that only about half of the protein-protein interactions have been identified [39]. The explicit representation of all molecular species and interactions in the network provides a clear and transparent view which is needed for applications working with multiple molecular –omes. At this stage, the reconstruction excludes transcriptional regulation because no database describes such genome-wide knowledge on the regulation functions that would be needed in the damage estimation and the reconstruction validation. The addition of this type of knowledge would be a useful next step towards a more complete network reconstruction. Another step towards a more complete reconstruction would also be the integration of RefRec with the consensus reconstruction of yeast metabolism [19]. The integration of the manually curated and continuously updated consensus network with RefRec would improve the quality of the metabolic network in RefRec and provide additional features for the consensus network.

Unique identification of objects is required for any large-scale integration. RefRec makes use of the reference object approach in which genes, transcripts, and proteins are identified using the knowledge of their primary sequence while metabolites and protein complexes are imported from single data sources. As an example of the importance of reference objects for large-scale data integration and production of coherent reconstructions, we note the extent to which the metabolic reconstructions KEGG and iND750 diverge (e.g., 22% of the metabolites in iND750 could not be matched with the metabolites in KEGG using comparisons of identifier strings), and how the recent community effort also utilizes the reference object approach to produce the consensus reconstruction that encapsulates previous metabolic reconstructions of yeast [19]. In the future, it is highly desirable that the reference approaches become more common as they provide a framework solid enough to large-scale integration of databases and reconstructions, as well as to cope with the growing amount of molecular data.

The global molecular interaction network reconstruction RefRec is freely available in the BioModels database in the Systems Biology Markup Language format (SBML) [40],[41] allowing additional analyses (see http://sbml.org to browse a large number of software supporting SBML). The format exploits Minimum Information Requested in the Annotation of biochemical Models (MIRIAM) [42] which is used to link the molecular species in RefRec to respective stable identifiers of the original source databases. This allows for unique identification of the objects in the databases and search of their additional features.

The comprehensiveness of RefRec provides potential for various analytical tasks. For example, we examined the portion of metabolism within the entire molecular interaction network, and estimated metabolic damages caused by gene knockouts. We found that metabolism constitutes 4.7% of the network, and the fraction is about the same if the KEGG metabolic reconstruction is replaced by the iND750 metabolic reconstruction (i.e., 4.6%, see the statistics of non-unique objects for iND750 in Table 4). A greater number of metabolic reactions are enzyme catalyzed in KEGG than in iND750 (2034 vs. 1063), and thereby the damage frequency of the metabolic reactions of KEGG was twofold in comparison to iND750. However, the flux balance based damage estimation method used for iND750 predicted five times larger damages in metabolism than the Boolean method used for the KEGG reconstruction (see Table 2). The difference is explained by more rigorous constraints applied by the flux balance method. The iND750 reconstruction brings additional value for the damage analysis by the means of compartmentalization of metabolites and reactions into distinct subcellular locations. While the KEGG reconstruction assumes all metabolic pathways to locate in a single cellular space, the iND750 reconstruction represents 8 compartments whose pathway damages can be studied separately. The explicit modeling of transport reactions between the compartments and exchange reactions between the intra- and extra-cellular spaces facilitates the iND750 reconstruction to simulate various cultivation conditions. In this study, we assumed a complex medium and aerobic cultivation in order to estimate the unconditional damages of gene knockouts. This made it possible to study a large number of experimental gene knockouts – however it occurred at the expense of producing more accurate conditional estimates on the damages.

**Table 4:** Number of unique molecular species and interactions in the iND750 metabolic reconstruction.

	Gene	Transcription	Transcript	Translation	Protein	Protein complex assembly interaction	Protein complex	Metabolic reaction	Metabolite
Number of objects	750				711			1,489 (1,812)	646 (1,177)
**Molecular species total**									2,115 (2,646)
**Interactions total**									1,489 (1,812)
**Objects total**									3,604 (4,458)

In parenthesis is given the number of non-unique objects, i.e., a unique object is counted as often as it exists in different compartments.

The unconditionally essential molecular species and interactions were determined based on the unconditional damage estimates of experimentally validated gene knockouts. About 1.7% of the studied knockouts were lethal and, out of them, 16.1% produced a blockage in the metabolism while 97.3% blocked protein-protein interactions. The result highlights the importance of the protein-protein interaction network for cellular viability. The calculated essentiality score suggested 689 unconditionally essential genes whose knockout always resulted in the inviable phenotype in the studied experimental context. These genes were primarily involved in central biological processes operating with all types of molecular species (genes, transcripts, proteins, metabolites, and protein complexes). This suggests that a reconstruction should involve these processes in order to produce comprehensive growth phenotype predictions. Specifically, the reconstruction should represent the needed molecular species and interaction types.

The notable difference in the number of unconditionally essential metabolic reactions in the KEGG vs. iND750 metabolic reconstruction reflects the properties of the two alternatives and their analysis methods. The iND750 reconstruction contains more than 600 metabolic reactions for which the flux balance method cannot determine any flux under any condition. These reactions are constitutively blocked by pathway dead-ends that take place because of gaps in the current understanding (e.g., erroneous or missing gene annotations), or simple decisions about the reconstruction boundaries. Although the flux balance method did not enable the damage estimation for the constitutively blocked reactions of iND750, it was possible to estimate the corresponding damages with the KEGG reconstruction and the Boolean method. For example, the Boolean method correctly predicted the essentiality of the alanine-tRNA ligase catalyzed reaction (needed in protein synthesis) while the corresponding reaction was constitutively blocked in iND750 and there caused failed predictions ([16], Supplementary material). The difference in the number of unconditionally essential reactions in the two metabolic reconstructions may also be explained by the damage sizes the analysis methods estimate for metabolism. As presented in Table 2, damages reach metabolic reactions more often but they block a smaller number of objects in the KEGG reconstruction than in the iND750 reconstruction. For the KEGG reconstruction this implies that reactions blocked by lethal knockouts are rarely blocked by viable knockouts, thereby giving them a high essentiality score. Thus, the predicted unconditionally essential reactions possibly include reactions that would not appear unconditionally essential in a closer examination.

The RefRec reconstruction was successfully validated using predictions of cellular inviability. The applied computational predictor correctly predicted most of the inviable phenotypes based on the damage estimates of the gene knockouts. The damages in the protein-protein interaction network had a greater effect on the phenotype prediction than the damages in the metabolic network as was shown by the exclusion of the protein-protein interaction network which dramatically reduced the number of correctly predicted inviable phenotypes. This might simply be due to the much higher coverage of the genome for the protein interaction network than for the metabolic network. Another holistic view to the reconstruction and its predictive performance was given through the Gene Ontology. As the RefRec reconstruction represents all the yeast genes, it also involves all the biological processes described in Gene Ontology. The comprehensiveness of RefRec was illustrated by the phenotype predictions which were found relatively independent on the examined processes that widely covered different molecular -omes and cellular phenomena. Altogether, the presented global reconstruction for the yeast molecular interaction network provides a sound foundation for further expansion and refinement, as well as for various systems biology approaches exploiting it. For example, the reconstruction may promote synthetic biology and strain optimization approaches by describing systemic context for the examined phenomena and providing integration facility for data from heterogeneous sources.

## Materials and Methods

### Building the RefRec reconstruction

Medicel Integrator software system (version 2.5, Medicel, Ltd., Espoo, Finland) and its comprehensive data schema were used for consistent integration of the information from the molecular databases presented in Table 1.

A unique set of molecular species was obtained from the source databases. Genes and transcripts were obtained from the Ensembl database while Ensembl, RefSeq and UniProt were used as the source databases for proteins. Within all three types of molecular species, primary sequences were used to identify the objects and, thereby, redundant objects were merged if they had 100% sequence identity (nucleic acid sequence from the first to the last exon of genes, nucleic acid sequence of coding sequences of transcripts, and amino acid sequence of proteins). As an example, the numbers of internally redundant gene, transcript, and protein entries in Ensembl database were 0, 50, and 80, respectively. Protein complexes were instantiated based on protein-protein interactions described in the IntAct database. Their identification was based on the identifiers of IntAct, as well as its cross references to the UniProt protein database. Metabolites were obtained from the KEGG GLYCAN and KEGG COMPOUND databases, and they were identified using the namespace of KEGG. Thus, the identification of protein complexes and metabolites relied on the namespaces of single databases. In the SBML representation of the network, all the molecular species are linked to their respective source databases using the recommended stable identifiers of the respective databases and the MIRIAM recommendations [42].

The set of the unique molecular species were connected by interactions. The reconstruction contains four types of interactions (transcriptions, translations, metabolic reactions, and assemblies of protein complexes) of which transcriptions and translations are not explicitly represented in any of the used source database. Therefore, a transcription was instantiated for each of the genes and a translation was instantiated for each of the transcripts. Metabolic reactions were obtained from the KEGG REACTION database which provides the knowledge about the substrates, the products, and the Enzyme Committee (EC) numbers for the metabolic reactions. EC numbers were used to connect the metabolic reactions with proteins having the respective catalytic activity according to the Swiss-Prot ENZYME database [43]. A separate metabolic reaction was reconstructed for each of the proteins capable for catalyzing a specific biochemical transformation. Reversible metabolic reactions were represented as two irreversible reactions in opposite directions. The IntAct database provided the knowledge for the protein complex assembly interactions. The assembly interactions were assumed to be irreversible, i.e., no respective disassembly interactions were reconstructed.

### Phenotype data on yeast mutants

Growth phenotypes of genetically mutated yeast strains are studied in several large-scale experiments in order to assess the essentiality of single genes (see, e.g. [21],[22]) and to explore phenomena related to knockouts of multiple genes (see, e.g. [35],[36]).

The phenotypic data on experimentally studied yeast mutants was obtained from three sources which provided partially overlapping information. First, the phenotypic information of single gene deletion mutants originates from [22]. The second source of phenotypic data was a set of large-scale experiments conducted using a synthetic genetic array [35],[36]. In these experiments, a large number of strains carrying a double gene mutation are produced by crossing mutations in 132 query genes into a set of ∼4,700 gene deletion mutants. In addition, these experiments describe the viability of a few hundred triple mutation strains. The third source, Saccharomyces Genome Database (SGD, http://www.yeastgenome.org/, accessed March 27^th^ 2008) summarizes numerous experimental results from original publications including the aforementioned data sources [22],[35],[36]. The SGD database was queried for synthetic genetic interactions including: synthetic lethal interactions where a double gene deletion or mutation causes an inviable mutant; synthetic growth defect interactions where a double gene deletion or mutation causes slow growing mutants; and synthetic rescue interactions where a gene mutation or deletion rescues the lethality or growth defect of a strain which is mutated or deleted for another gene. Table 3 summarizes numbers of mutated strains. The damage estimates, the essentiality score, and the computational classification were based on the data in Table 3.

### Boolean model and its analysis

The global molecular interaction network reconstruction was translated into a Boolean model which makes it possible to estimate the blocked molecular species and interactions and, thereby, the damage the gene knockouts cause to the molecular network. In the Boolean model, the possible blockage of each of the objects is represented by a Boolean-valued feasibility variable. The feasibility describes whether a molecular species exists in its functional form (Boolean value ‘True’) or not (‘False’), and, whether an interaction may occur (‘True’) or not (‘False’). The feasibility of an object is determined using an associated Boolean rule. The rules for interactions are based on molecular species required by the interaction (e.g., substrates and enzymes in the case of metabolic reactions, and transcripts in the case of translations). Thus, the feasibility *F* of an interaction *I* is determined as 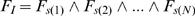 where 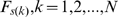 are the feasibilities of the molecular species needed by *I*, and ‘
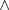
’ is the logical ‘and’ operator. On the other hand, the rules of the molecular species are based on the interactions producing them. The feasibility of a molecular species *S* is determined by the rule 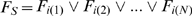 where 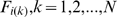 are the feasibilities of the interactions producing *S*, and ‘
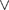
’ is the logical ‘or’ operator.

Gene knockout damages were estimated in the Integrator software system by simulating the constructed set of Boolean rules. A knockout was introduced by setting the given gene infeasible while all the other molecular species and interactions were set feasible. The rule set was then evaluated until all the feasibility values were fixed. The size of the final set of infeasible objects quantified the damage of the knockout. The approach has similarities to the methods used by [44],[16],[45].

### Metabolic reconstruction iND750 and its analysis

In order to exploit a constraint-based analysis method for the damage estimation in metabolism, we performed a joint analysis with RefRec and the metabolic reconstruction iND750 [16]. The iND750 reconstruction was used to replace the KEGG metabolic reconstruction in RefRec. By changing a single data source to another, we avoided discrepancies between the two sources, e.g., different metabolite naming conventions between KEGG and iND750.

Table 4 presents statistics for the genome-scale metabolic reconstruction iND750. Major differences to the KEGG reconstruction include the compartmentalization to 8 compartments in which the species and interactions are located, as well as transport reactions between the compartments and exchange reactions between extra- and intra-cellular spaces. In the statistics, reversible metabolic reactions are considered as two irreversible reactions in opposite directions which makes Tables 1 and 4 comparable. The iND750 reconstruction does not explicitly model transcriptions, transcripts, translations, protein complex assembly interactions, or protein complexes. Instead, they are modeled using gene-protein-reaction (GPR) associations.

The metabolic damage was estimated using a combination of Boolean reasoning and a flux balance based method. The GPR associations were interpreted as Boolean rules whose evaluation indicate whether a gene knockout causes the lack of an enzyme and thereby the blockage of the respective metabolic reaction [16]. A flux balance based method [33] was used to detect additional metabolic reactions which were blocked, i.e., they could not carry a flux under the given conditions. The blockage of metabolites was determined based on the calculated blockage information of the metabolic reactions and the contents of the cultivation medium. A metabolite was considered blocked if all the reactions producing it were blocked and its uptake was not possible from the cultivation medium.

In order to estimate unconditional metabolic damages (i.e., the objects blocked under all cultivation conditions), we assumed complex cultivation medium and aerobic conditions. Further, we assumed the capability of the cell to uptake and secrete all the metabolites for which the respective exchange reactions exist. The reactions blocked under these conditions are blocked under all other conditions, too [33]. The calculations were performed using MATLAB (version 2008b, The MathWorks, Inc., Natick, MA, US) and lp_solve (version 5.5, http://lpsolve.sourceforge.net/).

### Classification of growth phenotypes

The lethality of the ∼590,000 experimentally validated single, double, and triple gene knockouts was predicted using their damage estimates. As the damage was quantified by the Boolean-valued feasibility for the molecular species and interactions, the input data for the classification was a binary matrix with ∼590,000 rows (i.e., samples) and ∼67,000 columns (i.e., variables).

The feasibility data was preprocessed by excluding redundant information. If two or more molecular species or interactions (*i* and *j*) had the same feasibility under all the *N* knockouts 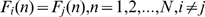, the variables were merged and considered as a single variable 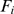. Typically, this merged the corresponding genes, transcriptions, transcripts, translations, and proteins together because of their direct relations on protein synthesis pathways. The preprocessing reduced the number of the variables from 67,288 to 18,366 without using the phenotype information to be predicted.

Prior to each classification, the samples were randomly divided into training and testing sets. In the case of Figure 8, the training samples always contained 1,400 lethal and 5,600 non-lethal knockouts. With the help of the training samples, the number of variables was further reduced by choosing feature variables for the classifier training. A variable *i* was used as a feature variable if its essentiality score 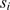 was greater than 0.5 in the training set. The essentiality score was calculated as 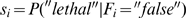, i.e., the probability of an inviable phenotype given the variable *i* was infeasible. The feature selection typically extracted ∼4,000 feature variables from the set of 18,366 variables.

Eventually, the selected training samples (7,000 in the case of Figure 8) were represented as binary vectors whose elements described the feasibility for the ∼4,000 feature variables (i.e., selected molecular species, interactions, and their merged sets formed during the preprocessing step). The training samples, associated with their corresponding experimental phenotype knowledge, were used to train a maximum margin support vector machine classifier (a MATLAB implementation of a learning-based algorithm for binary classification) [46],[47] to predict the knockout lethality. In our case, the computational complexity of the classification problem limited the maximal number of training samples to 7,000. The classifier determined an optimal hyperplane in the ∼4,000 dimensional Boolean space to separate the viable and the inviable samples. The samples reserved for the testing were subsequently used to assess the classifier performance. For each of the test samples, the classifier calculated the corresponding ∼4,000 dimensional binary vector and checked at which side of the hyperplane the vector laid.

## Supporting Information

Table S1 Unconditionally essential genes and their enrichments in GO Biological Processes.
(0.10 MB XLS)
